# Cancer Wars: Revenge of the AMPs (Antimicrobial Peptides), a New Strategy against Colorectal Cancer

**DOI:** 10.3390/toxins15070459

**Published:** 2023-07-14

**Authors:** Mina Răileanu, Mihaela Bacalum

**Affiliations:** Department of Life and Environmental Physics, Horia Hulubei National Institute of Physics and Nuclear Engineering, 30 Reactorului Street, RO-077125 Magurele, Romania; mina.raileanu@nipne.ro

**Keywords:** cancer therapy, Melittin, Cecropin A, colon carcinoma, Cecropin A—Melittin, spheroid

## Abstract

Cancer is a multifaceted health issue that affects people globally and it is considered one of the leading causes of death with a high percentage of victims worldwide. In recent years, research studies have uncovered great advances in cancer diagnosis and treatment. But, there are still major drawbacks of the conventional therapies used including severe side effects, toxicity, and drug resistance. That is why it is critical to develop new drugs with advantages like low cytotoxicity and no treatment resistance to the cancer cells. Antimicrobial peptides (AMPs) have recently attracted attention as a novel therapeutic strategy for the treatment of various cancers, targeting tumor cells with less toxicity to normal tissues. The aim of the study was to discover alternate treatments that do not lead to cancer resistance and have fewer side effects. Here, we report the effects induced by several AMPs, Melittin, Cecropin A, and a Cecropin A—Melittin hybrid, against two human colorectal cancer-derived spheroids. To study the effects of the peptides, cell viability was investigated using MTT, LDH, and ATP assays. Furthermore, cellular senescence and cell cycle were investigated. We found that using different concentrations of these peptides affected the spheroids, their structure being highly compromised by reducing cell viability, and the increase in ATP and LDH levels. Also, the cells are arrested in the G2/M phase leading to an increase in senescent cells. We show that Melittin and the hybrid are most effective against the 3D colorectal cancer cells compared to Cecropin A.

## 1. Introduction

Due to its high mortality rate, cancer treatment remains one of the biggest challenges in the public health system globally [1,2]. Most used therapeutic strategies include surgery, radiotherapy, chemotherapy, or a combination of these treatments. They are somewhat efficient in prolonging a patient’s life expectancy [3]. Unfortunately, several obstacles affect or limit their benefits. One such obstacle is drug access, restricted to the whole tumor volume due to the complexity and heterogeneity within the tumor or the surrounding microenvironment that leads to chemotherapy resistance [1]. That is why the efficiency of cell cultures used has been questioned in the last few years because of the impediments it could bring to the results obtained.

An important characteristic is related to the culture geometry, which up to recently, for cancer research studies, has involved two-dimensional (2D) cell cultures, which proved to be less reliable predictors for treatment response in vivo [4]. In a 2D cell culture model, the cells are grown flat, with limited neighboring cell contact and communication, as well as a larger surface exposed directly to nutrients and oxygen as compared to the in vivo system [5,6,7]. Using three-dimensional (3D) tumor spheroid cultures, we can overcome these issues. Tumor spheroid architecture allows the cells to develop more cell–cell contact and enhances inter-cellular communication [8]. Thus, the 3D model proves to be more accurate in drug treatment predictions as compared to 2D cultures [5].

Another undesirable characteristic comes from the drug’s lack of specificity, which can lead to healthy cell toxicity [9]. However, there is a need to develop more efficient drugs with less toxicity for healthy cells and more specificity for cancer cells [10]. A new class of natural and synthetic molecules which could overcome classic treatment shortcomings is antimicrobial peptides (AMPs), particularly the cationic peptides [11]. These molecules are found in various species like bacteria, fungi, invertebrates, vertebrates, and plants as part of the host’s innate immune system [12,13]. AMPs vary in length, ranging from a few amino acid residues up to 100, and they possess broad-spectrum antimicrobial activities against pathogens like bacteria, viruses, fungi, and protozoa [11,12]. Beside the antimicrobial properties of AMPs, recent studies have focused on their anticancer activity [11,14,15]. Thus, from the total of almost 10,000 peptides identified and reported in the DBAASP (Database of Antimicrobial Activity and Structure of Peptides), around 1400 also have anticancer activity [16].

In a previous study, we achieved improved intra-spheroid delivery and distribution of systemically administered doxorubicin (Dox) and gramicidin A (GA), a well-known antimicrobial peptide (AMP), within and on the spheroid periphery [17].

The anticancer effects of AMPs against colorectal cancer cells were previously studied, with several action mechanisms ranging from cell cytotoxicity (BmKn2 scorpion venom peptide [18]), reduction of the xenograft colorectal tumor cell mass in the zebrafish model (microcin E492 bacteriocin [19]), suppressed metastatic progression (nisin [20]) to the alteration of cell-cycle-regulatory proteins (bovine lactoferrin and LfcinB [21] or the metabolic profile (FF/CAP18 [22])). Several other natural and synthetic peptides show a high affinity for other types of cancer cells as well [23]. Melittin, the main active component of bee venom, is a 26-amino acid cationic and amphipathic peptide, with known antimicrobial, antitumoral, antifungal as well as antioxidant activity in vitro and in vivo [24,25]. Although Melittin’s mechanism is still under debated, it is widely accepted that it is a membrane-active peptide that, following membrane insertion, can drastically disrupt its integrity [26]. Another peptide used in our study, Cecropin A, is a 37-amino acid α-helical cationic and amphipathic peptide isolated from insects, which directly affects the microbial membrane by pore formation [27]. Cecropin A shows high affinity for both *Gram-positive* and *Gram-negative* bacteria and cancer cells, killing them by apoptosis or inducing cell cycle arrest; however, no lysis was observed in blood cells at similar concentrations [28]. The synthetic peptide derived from Cecropin A (1–7 residues) and Melittin (2–9 residues) proved to be more active against bacterial cells, with less hemolytic activity compared to the initial peptides [29]. The 15-amino acid hybrid showed anticancer activity against murine melanoma cells by translocating into cells and causing ATP synthesis inhibition and mitochondrial dysfunction, resulting in cell breakdown [30]. A recent study also reported on p53 plasmid delivery by the stearic acid (C18)-conjugated hybrid, which lead to the inhibition of cancer cell proliferation [31].

Colorectal cancer is considered to be the second most common adult cancer in women and the third most common in men. Worldwide, it is the fourth type of cancer and has accumulated 6% deaths globally [32]. The heterogeneity of colorectal carcinomas comes from the various gene mutations such as oncogenes, DNA repair and tumor suppressor genes, which makes the treatment difficult [33]. Considering these factors, using AMPs alone or combined with conventional chemotherapeutic agents can be seen as a new approach with possibly better impacts on colon cancer research and treatment [17].

Thus, the aim of this study was to further investigate the effects of three AMPs (Melittin, Cecropin A and a hybrid of Cecropin A and Melittin) against colorectal cancer cells spheroids (HT-29 and HCT-116). Spheroid morphology and evolution after peptide treatment was investigated by light microscopy. Further, cell viability was investigated using MTT, LDH and ATP assays. Finally, cellular senescence and cell cycle were studied. All three peptides investigated affected the spheroids by reducing cell viability and ATP, and increasing LDH levels, senescence and G2/M cell arrest. We show that Melittin and the hybrid are most effective against the 3D colorectal cancer cells compared to Cecropin A.

## 2. Results

### 2.1. Treatment Effect on Tumoral Spheroids Morphology

To determine the morphological effects of the cationic peptides, we conducted a treatment over time by applying various concentrations (1, 2.5, 5 and 10 µM) of AMPs (Melittin—Mel, Cecropin A—CA and Cecropin A-Melittin hybrid—CA-Mel) on spheroids that were formed with HT-29 and HCT-116 cells. The spheroids were seeded in low adhesion plates, and images of their evolution were taken with the help of a light microscope, at 24 and 48 h after peptide treatment, with the 4× objective.

**Figure 1:**
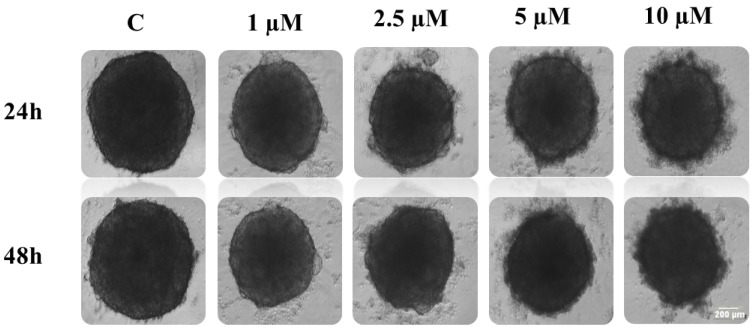
The cytotoxic effects of Mel on HT-29 spheroids. The spheroids were obtained after seeding 5000 cells/well and they were incubated for three days. On the third day of growth, the spheroids were treated with various concentrations of Mel (1, 2.5, 5 and 10 µM). Images were taken with the help of light microscope at 24 and 48 h, with the 4× objective. The scale bar is 200 µm.

**Figure 2:**
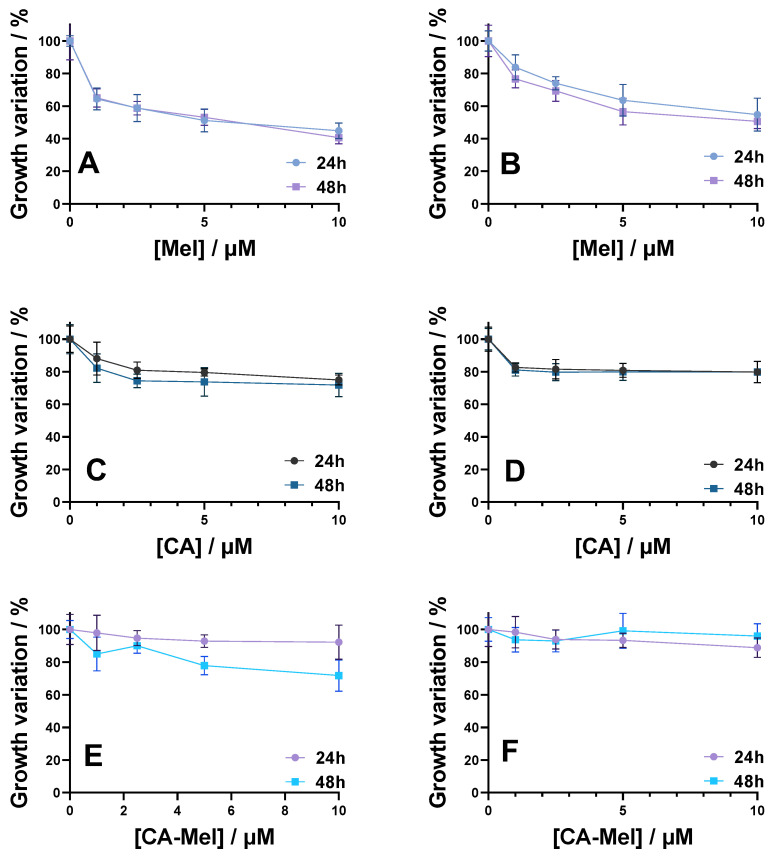
Evolution of HT-29 (**A**,**C**,**E**) and HCT 116 (**B**,**D**,**F**) spheroids treated for 24 and 48 h with Mel (**A**,**B**), CA (**C**,**D**) and CA-Mel (**E**,**F**). Data are represented as mean ± SD (*n* ≥ 3).

Following Mel treatment, the HT-29 spheroid’s integrity is affected with increasing peptide concentration (Figure 1). In comparison with the control spheroids, treated spheroids begin to lose mass and dead cells become detached, forming a halo of debris surrounding the spheroid body. This phenomenon is more visible from the second concentration of peptide (2.5 μM), with the most pronounced effect at the highest concentration tested (10 μM). The same effects were observed for both time conditions (24 and 48 h). When quantified, there is a significant reduction in spheroid size, up to 65% in comparison with the control spheroid, from the first concentration added, down to 45% at the last concentration of Mel after 24 h of treatment and 40% after 48 h (Figure 2A).

Treatment with antimicrobial peptide CA leads to significant morphological modifications of the HT-29 spheroids correlated with the increase in peptide concentration (Figure S1). Compared with the untreated spheroids, we can observe the spheroids’ alteration and vesicle formation. This phenomenon can be seen from the lowest concentration for all conditions investigated. Compared to Mel treated-spheroids, the mass reduction is observed form the first concentration and is only of 26% at the highest concentration after a 24 h treatment and 29% after a 48 h treatment (Figure 2C).

Finally, when CA-Mel treatment was applied, it led to less structural modifications (Figure S2). In this case, we can see an increase in the rate of compaction of the spheroids and the expansion of the necrotic core close to the proliferative layer. This phenomenon can be observed for all conditions investigated. The effect of CA-Mel is less pronounced; after 24 h, the mass of spheroids is reduced only by 8% for the highest concentration. A more pronounced effect is observed after 48 h of treatment with a decrease in mass of 29% (Figure 2E).

In the case of the HCT-116 spheroids, the treatment with the Mel leads to a loss of structural integrity of the spheroids, and an increase in the number of necrotic cells, all of these phenomena being correlated with the increase in peptide concentration (Figure S3). Similar to Mel-treated HT-29 spheroids, we can observe the formation of a halo comprised almost entirely from cellular debris. At the 2.5 μM concentration mark, the spheroids begin to destabilize and dead cells become detached. Similar to HT-29 spheroids, when HCT-116 spheroids are treated with Mel, the mass of the spheroids reduces to 83% from the control at the first concentration applied for 24 h. The size of the spheroids decreases with increasing concentration down to 54% at the highest concentration applied (Figure 2B). When treated for 48 h, the size of HCT-116 spheroids decreases from 76% when treated with 1 µM down to 50% for spheroids treated with 1 µM (Figure 2B).

Unlike HT-29 spheroids, treatment with CA produces less structural modifications to the HCT-116 spheroids (Figure S4). Nonetheless, compared to the control spheroids, a slight reduction in dimension of the treated spheroids from the first concentration applied can be observed. The size of the spheroids decreases from around 82% when treated with 1 µM of CA down to 79% with no significant change between 24 h and 48 h of treatment (Figure 2D).

Finally, treatment with the hybrid CA-Mel did not produce major structural modifications, nor a change in the size of the spheroids (Figure S5). The spheroids’ mass did not decrease more than 10% for all experimental conditions (Figure 2F).

The treatment with the peptides produced different effects which can suggest that different mechanisms were involved for each peptide. Thus, subjecting the tumoral spheroids to Mel treatment leads to a higher level of structural destabilization for both cell lines, followed by CA and CA-Mel hybrid which only slightly affect the spheroids.

### 2.2. Cell-Viability Measurements

The viability of the 3D cell cultures treated with different concentrations of peptides for 24 h or 48 h was evaluated using the MTT test, and by quantifying the levels of released ATP and LDH.

#### 2.2.1. MTT Assay Results

**Figure 3:**
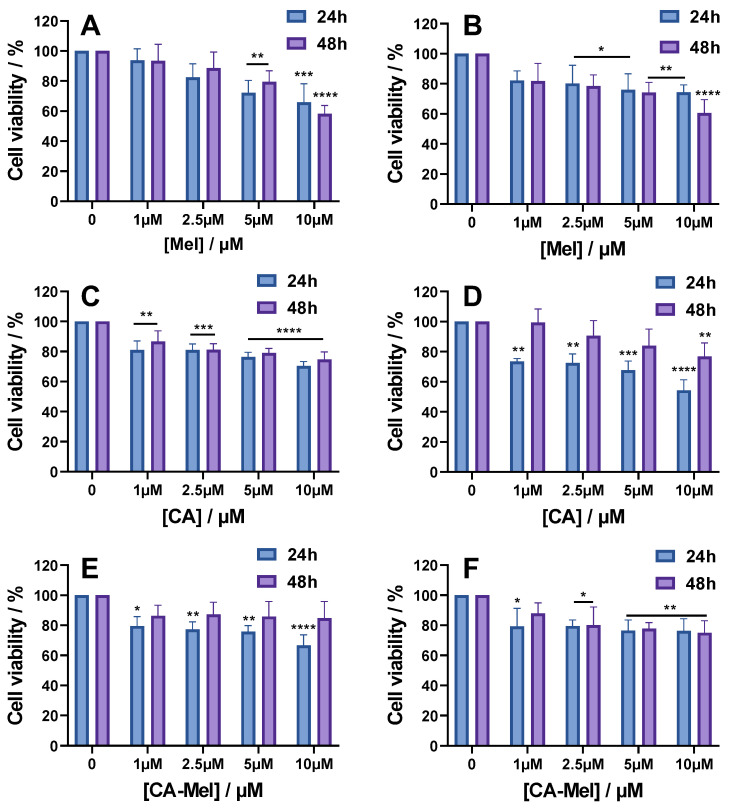
Cell viability of HT-29 (**A**,**C**,**E**) and HCT-116 (**B**,**D**,**F**) spheroids treated for 24 and 48 h with the following: Mel HT-29 (**A**,**B**), CA (**C**,**D**) and CA-Mel (**E**,**F**). Data are represented as mean ± SD (*n* = 3). *p* values were calculated using ANOVA analysis with Tukey’s multiple comparison post-test. * *p* < 0.5, ** *p* < 0.01, *** *p* < 0.001, **** *p* < 0.0001 vs. C.

The cell viability of HT-29 and HCT-116 spheroids, following AMPs treatment, are reported in Figure 3 for all experimental conditions investigated. For HT-29 spheroids treated with Mel for 24 h, cell viability decreases monotonously with the increase in peptide concentration. Thus, for 1 and 2.5 µM, the percentage of viable cells decreases to 93% and 82%, but the changes become significant when the spheroids were treated with 5 and 10 µM, decreasing the viability to 72% and 65%, respectively (Figure 3A). When the treatment is applied for 48 h, the only significant change compared to 24 h was seen at the last concentration used, which decreased the viability to 58% (Figure 3A). HCT-116 spheroids treated with Mel for 24 h or 48 h were less affected by the treatment compared to the HT-29 spheroids. Thus, cell viability decreased to 82% after the first concentration of peptide was used and was reduced to 74% when 10 μM Mel was used (Figure 3B). For spheroids treated with Mel for 48 h, cell viability was not greatly altered at low concentrations of the peptide compared to the treatment at 24 h, but a greater effect was observed at the concentration of 10 μM Mel where the viability dropped to 60% (Figure 3B).

For CA, the results indicate decreases in spheroid viability for both cell lines and treatment times, with increasing peptide concertation (Figure 3C,D). So, after HT-29 spheroids treatment for 24 h with 1 μM CA, cell viability decreased significantly to 81% and increasing peptide concentration up to 10 μM reduced the viability down to 70% (Figure 3C). When treated for 48 h with CA, the spheroid viability was affected in a similar manner to the treatment used at 24 h. For HTC-116 spheroids treated for 24 h with 1 μM CA, the cell viability decreased to 73% and continued to decrease down to 54% at the last CA concentration used (Figure 3D). Surprisingly, the cell viability for spheroids treated for 48 h with CA was slightly higher compared with the treatment applied for 24 h, but still decreased with increasing peptide concentration.

The last peptide studied was a hybrid of Mel and CA, CA-Mel. Similar to the results obtained for the other 2 peptides, the results indicate a decrease in viability with increasing peptide concentrations (Figure 3E,F). HT-29 spheroids treated for 24 h with 1 μM CA-Mel had a cell viability of 79% which continued to decrease to 66% at a concentration of 10 μM CA-Mel (Figure 3E). A longer treatment did not substantially change spheroid viability. HTC-116 spheroids treated for 24 h or 48 h with 1 μM CA-Mel had a cell viability of 79% and 87%, respectively, and continued to decrease to around 76% when treated with 10 μM CA-Mel (Figure 3F).

#### 2.2.2. ATP Assay Results

**Figure 4:**
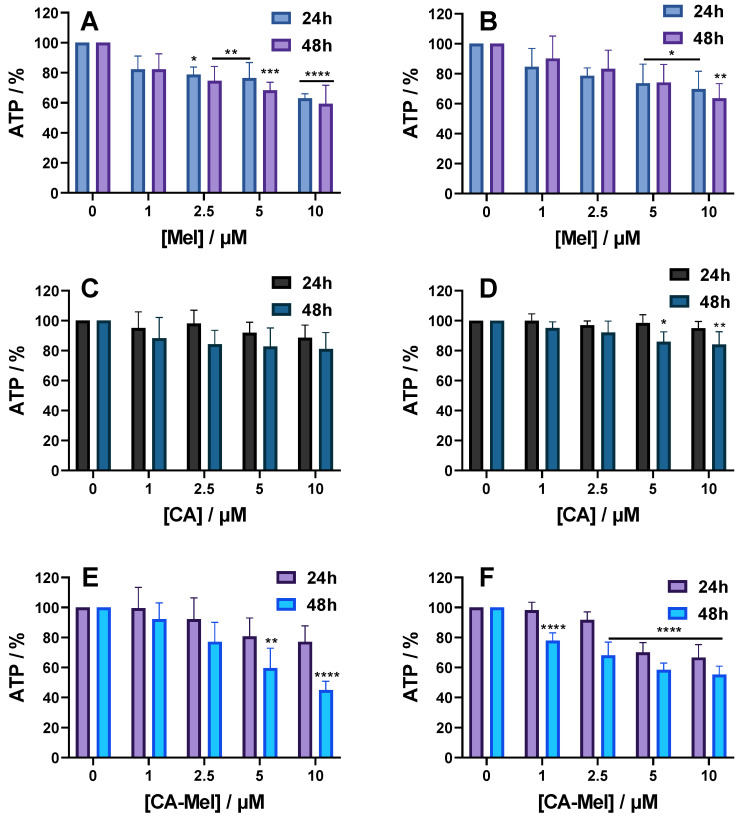
ATP level of HT-29 (**A**,**C**,**E**) and HCT-116 (**B**,**D**,**F**) spheroids treated for 24 and 48 h with the following: Mel HT-29 (**A**,**B**), CA (**C**,**D**) and CA-Mel (**E**,**F**). Data are represented as mean ± SD (*n* = 3). *p* values were calculated using ANOVA analysis with Tukey’s multiple comparison post-test. * *p* < 0.5, ** *p* < 0.01, *** *p* < 0.001, **** *p* < 0.0001 vs. C.

A second assay used to quantify changes in cell viability follows ATP levels in the cells after the treatment with peptides. For HT-29 spheroids treated with 1 μM Mel for 24 h and 48 h, the level of ATP decreased to 82% compared to control conditions. Starting from 2.5 µM, the ATP levels decreased significantly to 78% after 24 h and 74% after 48 h of treatment. Increasing peptide concentrations decreased the ATP level further, down to 63% after 24 h and 59% after 48 h of treatment at the highest concentration used (Figure 4A). For HCT-116 spheroids treated with 1 and 2.5 µM of Mel for 24 h, the level of ATP decreased slightly down to 84% and 78%, respectively. A higher effect was observed for the last two concentrations for which the ATP levels were reduced to 73% and 69% (Figure 4B). Similar to HT-29 spheroids, when treated for 48 h, no significant changes were found for ATP levels compared to 24 h.

CA treatment of HT-29 spheroids for either 24 h or 48 h led to a slight decrease, but no statistical difference was found between the experimental conditions. When treated with 1 μM, the ATP level decreased to 95% at 24 h and to 88% at 48 and continued to decrease to 88% and 81%, respectively, at the last concentration tested (Figure 4C). Treatment of HCT-116 spheroids for 24 h with 1 μM CA led to a decrease to 99% that continued to 95% of the ATP level, at the last concentration with no statistical difference (Figure 4D). However, when treated for 48 h, the ATP levels of HCT-116 spheroids were at 95% for the first concentration and decreased significantly to 84% for the last concentration tested.

For the last peptide investigated, HT-29 spheroids treated for 24 h with 1 μM of CA-Mel ATP level decreased slightly to 99% and decreased further down to 77% at the last concentration (Figure 4E). When treated for 48 h, the changes in ATP level were higher, with a monotonous decrease from 92% at the first concertation to 44% at 10 µM of CA-Mel. HTC-116 spheroids treated for 24 h with 1 μM CA-Mel had an ATP level of 98% which decreased to 66% when are with the last concentration (Figure 4F). Similar to HT-29 spheroids, when treated for 48 h, the effect increased, with ATP levels decreasing from 77% at the first CA-Mel concentration applied to 55% for spheroids treated with 10 μM CA-Mel.

#### 2.2.3. LDH Assay Results

**Figure 5:**
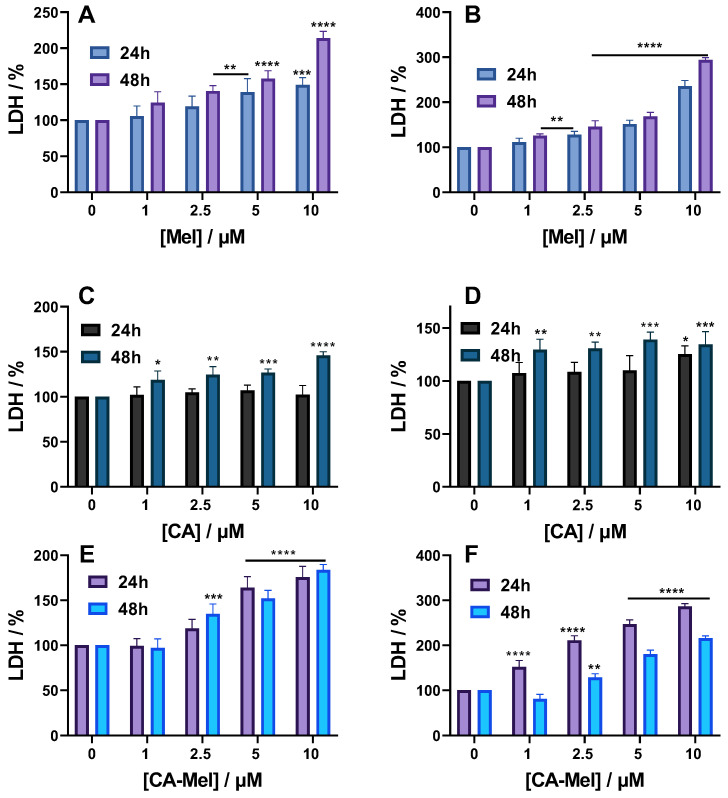
LDH level of HT-29 (**A**,**C**,**E**) and HCT-116 (**B**,**D**,**F**) spheroids treated for 24 and 48 h with the following: Mel HT-29 (**A**,**B**), CA (**C**,**D**) and CA-Mel (**E**,**F**). Data are represented as mean ± SD (*n* = 3). *p* values were calculated using ANOVA analysis with Tukey’s multiple comparison post-test. * *p* < 0.5, ** *p* < 0.01, *** *p* < 0.001, **** *p* < 0.0001 vs. C.

LDH release was the final parameter used to investigate how spheroids were affected by the treatment. When treated with Mel, the level of LDH in HT-29 spheroids increased monotonously from 105% at 1 μM to 148% at the last concentration tested (Figure 5A). Appling the treatment for a longer time increased the percentage of LDH released to 124% for the first concentration, up to 213% for 10 μM. When HCT-116 spheroids were treated with Mel for 24 h or 48 h, a higher percentage of LDH was released compared to HT-29 spheroids. Thus, when treated for 24 h, the LDH level at the first concentration of Mel applied was 111%, and increased with increasing concentrations up to 235% for 10 μM Mel (Figure 5B).

Treating HT-29 spheroids for 24 h with CA did not cause any significant changes compared to the control condition; the LDH level was at 102% for the highest concentration tested (Figure 5C). Appling CA for 48 h, however, has induced a release of 118% LDH for the first treatment concentration, up to 145% when 10 µM CA was applied. When HCT-116 spheroids were treated for 24 h with CA, a slight LDH increase of 107% was obtained for 1 μM of peptide. Increasing concentrations of peptide induced a larger LDH release, which became significant at 125% when 10 μM of CA was used (Figure 5D).

For the last peptide studied, a significant LDH release was observed for almost all experimental conditions tested. Thus, when CA-Mel was applied for HT-29 spheroids, the first concentration used did not affect the spheroids; the percentage of LDH released was 102%. However, increasing the peptide concentrations led to more LDH release, which became significant for the last two concentrations used with a percentage of 164% and 175%, respectively. A longer treatment will cause more LDH release with a significant percentage of 134% found after applying 2.5 μM. Increasing peptide concentration caused more LDH to release up to 183% at the final concentration used (Figure 5E). When HCT-116 spheroids were treated with CA-Mel, a stronger effect was observed, with significant LDH release for all conditions. After a 24 h of treatment with 1 μM CA-Mel, the LDH level was 152% and increased up to 286% at the last concentration applied (Figure 5F). Surprisingly, the LDH values are smaller after a 48 h treatment, but are still significant for concentrations higher than 2.5 µM, going above 200% at the last concentration tested (Figure 5F).

### 2.3. Cell Cycle Analysis

**Figure 6:**
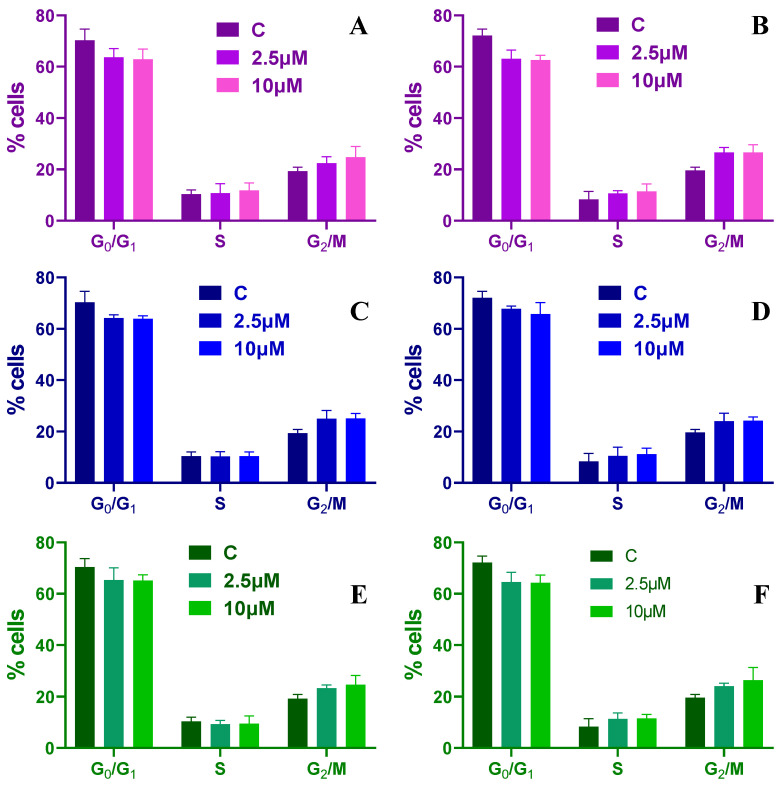
Cell cycle distribution for HT-29 spheroids treated with 2.5 and 10 µM of Mel (**A**,**B**), CA (**C**,**D**) and CA-Mel (**E**,**F**) for 24 h (**A**,**C**,**E**) and 48 h (**B**,**D**,**F**). Data are represented as mean ± SD (*n* = 3).

To further investigate the effects induced by the peptide treatment, cell cycle analysis was investigated at 24 and 48 h post treatment for two different peptide concentrations (2.5 and 10 µM). The results show that increasing antimicrobial peptide concentrations affects cell cycle progression. When HT-29 spheroids were treated with Mel the number of cells in S phase were not changing independent of the concentrations and treatment time compared with the control condition. However, after a treatment of 24 h, the cells in the G2/M phase increased from ~19% to ~22% for a concentration of 2.5 µM and to ~24% at 10 µM, while the number of cells found in G0/G1 phase decreased from ~70% to ~63% and finally to ~62% (Figure 6A). Finally, when HT-29 spheroids were treated for 48 h with Mel, the cells found in G2/M phase increased to ~26% for both peptide concentrations, while the ones found in G0/G1 phase decreased to ~62% (Figure 6B).

When HT-29 spheroids are treated with CA, the percentage of cells found in the G0/G1 phase decreases to ~63% and the ones in the G2/M phase increase to ~25% for the highest concentrations used independent of the treatment time applied (Figure 6C,D). Similar findings are found when the spheroids are treated with CA-Mel (Figure 6E,F). These results indicate that the peptide treatment induces a G2/M-phase arrest of the HT-29 cells.

**Figure 7:**
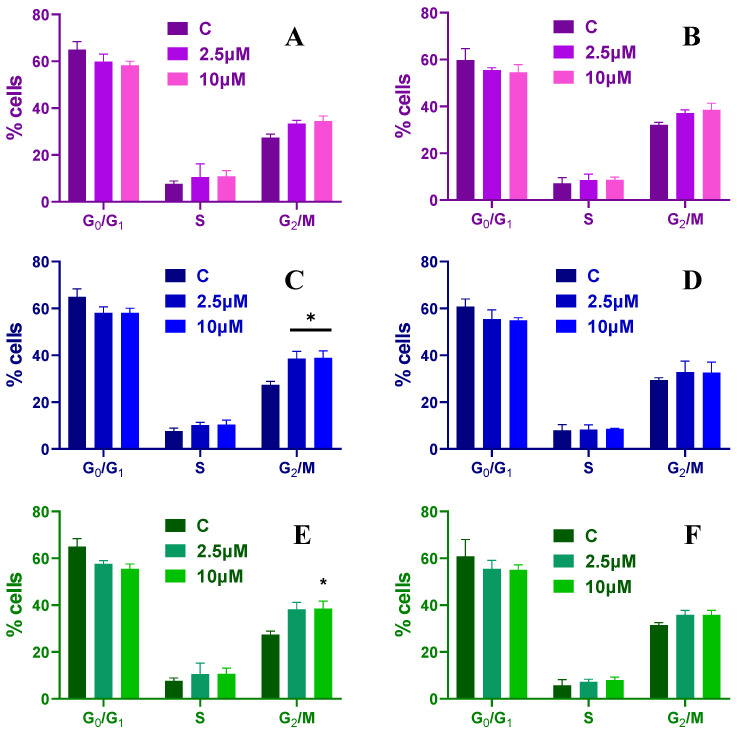
Cell cycle distribution for HCT-116 spheroids treated with 2.5 and 10 µM of Mel (**A**,**B**), CA (**C**,**D**) and CA-Mel (**E**,**F**) for 24 h (**A**,**C**,**E**) and 48 h (**B**,**D**,**F**). Data are represented as mean ± SD (*n* = 3). *p* values were calculated using ANOVA analysis with Tukey’s multiple comparison post-test. * *p* < 0.5 vs. C.

When HCT-116 spheroids are treated with Mel, the number of cells in the S phase increases slightly over time and different peptide concentrations, without any statistical difference. After a 24 h treatment with Mel, the cells found in the G0/G1 phase decrease from ~64% to ~59% for a concentration of 2.5 µM and to ~58% at 10 µM, while the number of cells found in the G2/M phase increases from ~27% to ~33% and finally to 34% (Figure 7A). When treated longer, the cells found in the G2/M phase increase to ~37% and ~38%, while the ones found in G0/G1 phase decrease to ~55% and 54%, respectively (Figure 7B).

HCT-116 spheroids treated for 24 h and 48 h with CA have an increase in the percentage of cells found in the G2/M phase up to ~38% and the ones found in G0/G1 phase are decreasing to ~58% for all concentrations used (Figure 7C,D). Similar results are obtained when the spheroids are treated with 2.5 and 10 µM of CA-Mel (Figure 7E,F). These results indicate that HCT-116 spheroids showed a significant increase in phase-locked G2/M cells upon peptide treatment.

### 2.4. Senescence Assay Results

**Figure 8:**
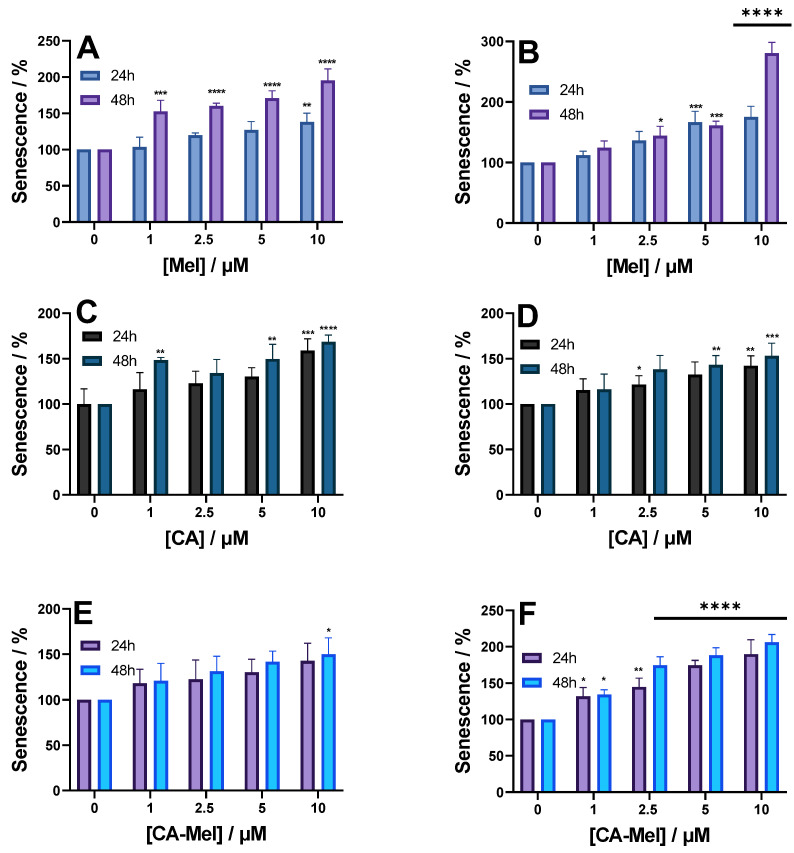
Senescence level of HT-29 (**A**,**C**,**E**) and HCT-116 (**B**,**D**,**F**) spheroids treated for 24 and 48 h with the following: Mel HT-29 (**A**,**B**), CA (**C**,**D**) and CA-Mel (**E**,**F**). Data are represented as mean ± SD (*n* = 3). *p* values were calculated using ANOVA analysis with Tukey’s multiple comparison post-test. * *p* < 0.5, ** *p* < 0.01, *** *p* < 0.001, **** *p* < 0.0001 vs. C.

Finally, we investigate the number of senescent cells after HT-29 and HCT-116 spheroid treatment with various peptide concentrations for 24 and 48 h (Figure 8). HT-29 spheroids treated with Mel for 24 h show a monotonous increase in senescent cells. When treated with 1 μM, the percentage of senescence was slightly higher at 103%, going up to 138% at the last concentration investigated (Figure 8A). When the same concentrations were applied for 48 h, the number of senescent cells increased significantly for all conditions. At the first concentration applied, the senescent cells increased ~1.5 times compared to the control condition (Figure 8A). For the following concentrations, the number of senescent cells increased slightly, up to ~1.8 when the spheroids were treated with 10 µM (Figure 8A). For HCT-116 spheroids treated with Mel for 24 h, senescence increased proportionally with increasing peptide concentration from 112% at 1 μM Mel to 175% at a concentration of 10 μM Mel (Figure 8B). Treating the HCT-116 spheroids for 48 h generated a similar number of senescent cells for the first peptide concentration compared to the 24 h treatment, except for the last concentration when the number of senescent cells increased by almost 3 times (Figure 8B).

Compared to Mel, when CA is applied to both spheroids, the results indicate smaller increases in senescence (Figure 8C,D). Thus, after treatment of HT-29 spheroids for 24 h with 1 μM CA, senescence increases to 116% and continues to increase up to 168% at the highest concentration applied (Figure 8C). Similar results were almost obtained when the peptide was applied for 48 h. Treating HTC-116 spheroids for 24 h with 1 μM CA increased the number of senescent cells to 115% and continued to increase up to 142% when treated with 10 μM CA (Figure 8D).

Similar to the results obtained for the other 2 peptides, the last peptide studied, CA-Mel, also increased the number of senescent cells (Figure 8E,F). When CA-Mel was applied to HT-29 spheroids, we saw a continuous increase in the number of senescent cells, from 118% when 1 μM CA-Mel was added for 24 h up to 142% at the last concentration (Figure 8E). The same results were obtained when the peptide was applied for 48 h. Subjecting HCT-116 spheroids to the peptide had a stronger effect compared to HT-29 spheroids. When treated with 1 μM CA-Mel, for both time ranges, the number of senescent cells increased by 1.3 times. For the following concentrations, the number increased by ~1.5 up to 1.9 times at the last concentration tested (Figure 8F).

## 3. Discussion

Colorectal cancer is ranked third among the most common cancer types worldwide, with almost 30% of new cases occurring annually [34]. The anatomical location at the pelvis level and the strategic supply from the circulatory and lymphatic system characterize colorectal cancer as a distinct entity concerning the invasive growth process, surgical approach, and effects of the treatments [35]. According to existing treatment strategies, patients with colorectal cancer undergo either long-term neoadjuvant radio-chemotherapy or short-term radiation therapy before completely removing the tumor [36]. Despite improved regimens, overall cure rates have not changed significantly, and most importantly, the response of individual patients to radio-chemotherapy, or radiation therapy alone, varies widely without predictability. Thus, it is of great clinical importance to implement a new model for predicting individual responses to various types of treatment. In order to investigate the individualized response to therapy, monolayer cell cultures are used extensively in many translational approaches. However, when the effects are induced by the treatment, there is a clear discrepancy between the results obtained in 2D and 3D cultures [37,38,39,40]. Spheroids have been shown in the literature to better simulate the in vivo tumor environment due to similarities in oxygen distribution, pH, nutrients, growth factors, cell signaling, and cell matrix organization [41].

Cationic peptide antitumor activity was wildly studied in 2D cultures. We previously investigated the effects of Mel and CA against the HT-29 cell line with an IC_50_ value in the range of 2–2.5 µM range for Mel and no IC_50_ value for CA at the concentrations tested [42]. An in vitro study led by Maher and McClean showed the degree of cytotoxicity of various antimicrobial peptides obtained from prokaryotic and eukaryotic organisms, such as galidermin (produced by *Streptococcus gallinarium*), nisin A, magainin I, magainin II, and melittin against colorectal cancer epithelial cells, HT-29 and Caco-2 [43]. Nisin A exerted a significant cytotoxic effect compared to galidermin on the two tumor cell lines, with galidermin having lower hemolytic activity compared to magainin I and II. The study showed that melittin was the most potent cytotoxic peptide (20 times higher toxicity) among the antimicrobial peptides tested, the effects being visible due to changes in cell morphology. It was concluded that melittin could exert its effect through several toxicity-signaling mechanisms, including cytolysis. The cytotoxic potential of eukaryotic antimicrobial peptides was found to be greater on colon cancer cells than that of peptides of microbial (i.e., prokaryotic) origin. These differences have been attributed to the different modes of action of these peptides on the target cell membrane [43]. Examples of peptides with significant anticancer effects are cecropins A and B that cause the direct lysis of tumor cells, most likely by disrupting the membranes of target cells. The lytic and antiproliferative activity of the cecropin class was restricted to cells becoming malignant, while benign fibroblasts were spared from its cytotoxicity. These properties were reported by Moore et al., being the first authors to describe the anticancer activity of cecropins [44].

The tumor spheroid model can be used in testing new chemotherapeutic agents; however, to our knowledge, there are only a limited number of studies reporting on antitumor peptides [17,45,46,47]. A recent study by Hadianamrei and collaborators has reported on the anticancer activity of short cationic a-helical peptides against both HCT-116 cells grown in a monolayer or spheroids [45]. Two of the peptides designed in the study (Cl-15 and Gl-15) showed a high effect against both 2D and 3D spheroids, with IC50 values obtained in the 2D system at 7.7 µM and 13.6 µM, respectively. Three other peptides reported in the study showed good results against 2D-cultured cells, with IC50 values between 15.6 and 29 µM; however, the effects against the HCT-116 spheroids were not significant. Four of the peptides had reduced toxicity against human dermal fibroblasts, which indicated that the use of lysine residues instead of arginine ones would reduce the cytotoxic effect on normal cells [45]. We also reported previously the antitumor activity of one known AMP, Gramicidin A [17], and a new synthetized peptide, P6 [46], with improved activity against HT-29 and HCT-116 spheroids, respectively.

Various tumor entities such as leukemia, lymphoma, colon carcinoma, lung and gastric cancer have been described to be sensitive to the lysis effect mediated by cecropin in vitro [48,49]. In our experiments, we found a similar trend of increased susceptibility of tumor cells treated with cecropin. However, this trend was not as significant as in the case of treatment with melittin or the hybrid, cecropin A-melittin. The cytolytic mechanism of antimicrobial peptides remains quite controversial. In addition, compared to the disruption of the membrane surface of tumor cells, which induces cytolysis/necrosis, a second mechanism of action involved in the destruction of tumor cells is the disruption of the mitochondrial membrane structure, which further leads to the activation of apoptotic pathways [50]. Cecropins and other antimicrobial peptides can be considered promising new chemotherapeutic agents because they demonstrate unique characteristics: their selectivity for malignant cells and their pronounced lytic activity on cancer cells allows for optimal therapy in vivo at low therapeutic concentrations and with limited side effects. The molecular basis for this antitumor activity of antimicrobial peptides has not been fully understood. Several studies have suggested that certain physicochemical properties of target cell membranes, such as differences in the number of lipoproteins present in the membrane or fluidity, may explain this phenomenon [51].

The research in this study is important because most current studies on tumor cell lines are still performed in 2D cultures. However, if these results will have to be transferred to a clinical study, it will become necessary to use 3D experimental models that more faithfully mimic the in vivo tumor microenvironment. Because of their structure, compact spheroids are more resistant to drug treatments than less-compact spheroids, where peptides can more easily diffuse to all regions of the spheroid, including their necrotic center. Spheroids larger in size than those derived from the HCT-116 cell line showed increased viability compared to HT-29 spheroids, indicating the presence of a heterogeneous cell population. It was observed that the treatment with antimicrobial peptides induces changes in the cell–cell interaction, in the proliferation area of the spheroids, leading to an increased penetration of nutrients towards the center of the spheroid, thus initiating the proliferation of passive cells (quiescent cells). It was shown that tumor spheroids, formed from HT-29 and HCT-116 cells, are more resistant to chemotherapy but had significant responses to the applied treatments. MTT analysis of HT-29 and HCT-116 spheroids, following peptide treatment, recorded a constant decrease in cell viability with increasing peptide concentration (most significantly in the case of Mel and CA-Mel peptides). In good correlation with the MTT, LDH levels, which are indicative of membrane permeabilization, are increasing significantly with increasing peptide concentration, with the best results obtained for the Mel and CA-Mel peptides. Also, a drastic decrease in ATP level was observed in Mel- and CA-Mel hybrid-treated spheroids compared to CA-treated spheroids. The results were expected considering that Mel has an increased toxicity to all types of eukaryotic or prokaryotic cells with no specificity for any cell type [42,52]. As reported previously [42], CA has a less toxic effect on cancer cells compared with Mel, which was also observed here. The hybrid between Mel and CA proved to have a more toxic effect compared to CA at the same concentration, but a similar concentration to Mel. This was to be expected considering that the hybrid obtained is desired to preserve the toxic capabilities of Mel. Looking further to the mechanism that can lead to cell death post treatment, we monitored the cell cycle progression. Normally, the cells follow the cell cycle until phase G2/M, where they should form two separate cells and then re-enter in the G0/G1 phase. However, due to DNA lesions induced by the peptide’s treatment, the cells are stuck in the G2/M phase, especially when treated with the highest concentration of 10 µM. The induction of cell cycle arrest at a specific checkpoint thereby inducing apoptosis is a common mechanism for the cytotoxic effects of anticancer drugs [53]. It has been reported that many anticancer agents arrest the cell cycle at then G0/G1, S, or G2/M phase and then induce apoptosis cell death [54,55].

Multiple types of stress can result in the same cases in the typical cellular senescence initiation, which is characterized by a metabolically active cell, arrested in one of the phases of the cell cycle, and can attain specific phenotypical alterations. Despite the fact that these cells sustain their metabolic activity and show resistance to apoptosis, which serves as an important role in maintaining the integrity of cell function, the dysfunctional effects that are intra- and extracellular in the case of cellular senescence are not to be ignored. In our study, the treatment with the peptides has increased the number of senescent cells, with the best results obtained for CA-Mel and Mel peptides, starting from the first concentration applied. Preclinical in vitro studies and in vivo trials have demonstrated that AMPs, alone or in combination with different treatment strategies, could lead to the development of an efficient and safe therapeutic alternative to present therapeutic regimens that are based on a high dose of nonspecific but also harmful cytotoxic agents [56].

More research is needed to demonstrate that cells in 3D cultures, particularly patient-derived spheroids, better reflect tumor morphology in vivo. Thus, the experimental and treatment regimen can be used to predict patients’ response to this type of therapy.

## 4. Conclusions

In the current study, we investigated the effects induced by the chemotherapeutic treatment of three anti-tumor peptides against HT-29 and HCT-116 tumor spheroids. Treatment with the peptides showed significant effects on HT-29 and HT-116 spheroids. All three viability assays showed that the peptides are decreasing the viability of the cells, due to membrane permeabilization and a depletion in the ATP levels, with the most significant results for the Mel and CA-Mel peptides. Also, when high concentrations of peptides were used (10 µM), the cells were arrested in the G2/M phase with an increase in the number of senescent cells, especially when treated with Mel and CA-Mel peptides. Further studies are needed to better understand, at the molecular level, the effects induced by the peptides in cancer cells. However, we can state that the peptides Mel and CA-Mel show promising therapeutic potential and can be taken into consideration for further studies involving the development of new anticancer therapies, either in combination with other drugs or incorporated into delivery systems in order to improve their action.

## 5. Materials and Methods

### 5.1. Materials

Antimicrobial peptides Melittin (GIGAVLKVLTTGLPALISWIKRKRQQ-NH_2_), CA (KWKLFKKIEKVGQNIRDGIIKAGPAVAVVGQATQIAK-NH_2_) and CA-Mel (CA (1–7) M (2–9)—KWKLFKKIGAVLKVL-NH_2_) were purchased from Bachem (Bubendorf, Switzerland). Dimethyl sulfoxide (DMSO) was purchased from Merck (Darmstadt, Germany), 3-(4,5-dimethylthiazol-2-yl)-2,5-diphenyltetrazolium bromide (MTT) was purchased from Serva (Heidelberg, Germany) and RNase, propidium iodide (PI) and Triton TX-100 were purchased from Sigma-Aldrich (Saint Louis, MO, USA). All cell cultivation media and reagents were purchased from Biochrome AG (Berlin, Germany).

### 5.2. Cell Culture

Human colorectal carcinoma cell lines, HT-29 and HCT-116 were purchased from ATCC (Virginia, USA), cultured in Dulbecco’s modified Eagle medium (DMEM), supplemented with 10% fetal bovine serum (FBS) and penicillin-streptomycin (0.5%–100 units/mL) (Biochrom). Cultures were maintained in a humidified atmosphere of 95% air/5% CO_2_ at 37 °C.

### 5.3. Spheroid Formation and Analysis

A seeding concentration of 5000 cells/well HT-29 and HCT-116 cells was used to form spheroids. A final volume of 200 µL of cell suspension was placed in each well of a clear, round-bottom, ultra-low attachment 96-well microplate (Corning^®^ 96-well Spheroid Microplates). After this, the plate was centrifuged for 1 min and then incubated at 37 °C for up to 5 days. The protocol is similar to the one used in a study conducted by Răileanu et al. [17]. Spheroid formation was confirmed by observing the plate under a light microscope (Olympus CX23 Binocular Microscope, Düsseldorf, Germany). Spheroids were monitored daily and the incubation medium was replaced every 3 days.

### 5.4. Treatment of the Tumoral Spheroids

Treatment evaluation was performed on the spheroids at 3 days post seeding by applying four concentrations (1, 2.5, 5, and 10 µM) of melittin (Mel), cecropin A (CA) and the hybrid cecropin A-melittin (CA-Mel). The changes in spheroid integrity were evaluated by light microscopy 24 and 48 h after treatment.

### 5.5. Cell-Viability Assays

#### 5.5.1. MTT Assay

The culture medium was removed from each well after the desired treatment times (24 and 48 h). MTT was added to each well at a final concentration of 1 mg/mL and the cell culture was further incubated. After 4 h, the medium was removed and DMSO was added to dissolve the crystals that had formed. Optical absorbance was recorded at λ = 490 nm using a Mithras LB 940 plate reader (Berthold, Germany). Cell viability was calculated using the following formula:

(1) $$\%\mathrm{viable}\;\mathrm{cells}=\frac{\left(\mathrm{Corrected}\;\mathrm{absorbance}\;\mathrm{of}\;\mathrm{treated}\;\mathrm{cells}\right)}{\left(\mathrm{Corrected}\;\mathrm{absorbance}\;\mathrm{of}\;\mathrm{control}\;\mathrm{cells}\right)}\;\;\;\times \;100$$

#### 5.5.2. ATP Measurements

ATP levels in the treated spheroids were assessed, as described briefly. Here, 100 µL of medium was removed from each well, then the remaining 100 µL with the spheroid was transferred into an opaque 96-well plate. After this, 100 µL of CellTiter-Glo^®^ reagent (Promega, Madison, WI, USA) was added onto the spheroids, which were incubated at room temperature for 10–15 min under thorough shaking to make sure that the spheroids were broken. Finally, the luminescence of the cells was measured using a Mithras LB 940 plate reader. The percentage of ATP level was estimated using the following formula:

(2) $$\%\mathrm{ATP}=\frac{\left(\mathrm{Corrected}\;\mathrm{luminescence}\;\mathrm{of}\;\mathrm{treated}\;\mathrm{cells}\right)}{\left(\mathrm{Corrected}\;\mathrm{luminescence}\;\mathrm{of}\;\mathrm{control}\;\mathrm{cells}\right)}\;\;\times \;100$$

#### 5.5.3. LDH Measurements

The LDH levels (Invitrogen™ CyQUANT™ LDH Cytotoxicity Assay) were recorded at two time points (24 and 48 h). From each sample medium, 50 µL was transferred to a 96-well flat-bottom plate. The plate was incubated at room temperature for 30 min and protected from light. After the incubation time, 50 µL of Stop Solution was added to each sample well, then mixed by gentle tapping. After the Stop Solution was added, the absorbance was measured within 1 to 2 h. The absorbance measured at λ = 490 nm.

### 5.6. Senescence Measurements

After the treatment (24 and 48 h), the spheroids were washed with PBS and fixated with a 2% paraformaldehyde solution for 10 min. The spheroids were washed in a 10% BSA solution in order to remove the fixation solution and then proceeded to stain the spheroids with the CellEvent™ Senescence Green Probe provided by the CellEvent™ Senescence Green Detection Kit (ThermoFisher Scientific, Waltham, MA USA) and were incubated for 2-and-a-half hours at 37 °C without CO_2_ and in the absence of light. After incubation, the spheroids were washed with PBS and the fluorescence was measured using λexc = 485 nm and λem = 535 nm with the Mithras LB 940 plate reader.

### 5.7. Cell Cycle Analysis

After 24 and 48 h of peptide treatment, the spheroids were harvested, trypsinized to detach the cells from the spheroidal shape and fixed with a pre-chilled 70% ethanol solution at −20 °C. Next, the cells were incubated with 0.2 mg/mL RNase and 20 μg/mL propidium iodide (PI) in a 0.1% Triton TX-100 solution in the dark for 30 min at 37 °C. Cell cycle distribution was analyzed by flow cytometry using a Beckman Coulter Cell Lab Quanta SC Flow Cytometer, 771917 Laser, Arc, MPL flow cytometer and data were analyzed using the Quanta Analysis software.

### 5.8. Statistical Analysis

Data are presented as the mean ± standard deviation (SD). Statistical analysis was performed using GraphPad Prism software (San Diego, CA, USA). The statistical significance of differences between experimental groups was calculated using one-way analysis of variance with Tukey’s multiple comparison test. The values of *p* < 0.05 were considered statistically significant.

## Data Availability

Not applicable.

## Supplementary Materials

The following supporting information can be downloaded at https://www.mdpi.com/article/10.3390/toxins15070459/s1: Figures S1–S5. The cytotoxic effects of CA on HT-29 spheroids; The cytotoxic effects of CA-Mel on HT-29 spheroids; The cytotoxic effects of Mel on HCT-116 spheroids; The cytotoxic effects of CA on HCT-116 spheroids; The cytotoxic effects of CA-Mel on HCT-116 spheroids.
