# Supplementary material for: Cancer Wars: Revenge of the AMPs (Antimicrobial Peptides), a New Strategy against Colorectal Cancer
Source: Toxins (Basel). 2023 Jul 14;15(7):459. doi: 10.3390/toxins15070459 (PMC10467133; doi:10.3390/toxins15070459)
Supplement: Supplementary file 1 [file toxins-15-00459-s001.zip › toxins-2476136-supplementary.pdf]

Supplementary Information

to

Cancer Wars: Revenge of the AMPs (Antimicrobial peptides), a new strategy against colorectal cancer

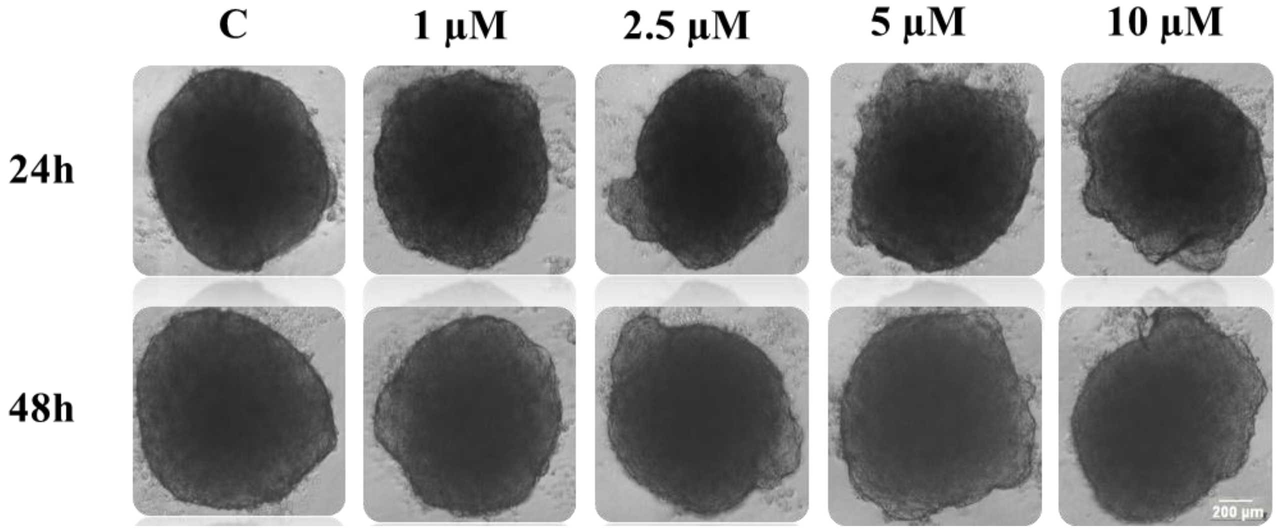

Figure. S1 The cytotoxic effects of CA on HT-29 spheroids. The spheroids were obtained after seeding 5000 cells/well and they were incubated for three days. On the third day of growth, the spheroids were treated with various concentrations of CA (1, 2.5, 5 and 10 μM). Images were taken with the help of light microscopy a 24 and 48 h, with the 4x objective. The scale bar is 200 μm and is identical for all the images.

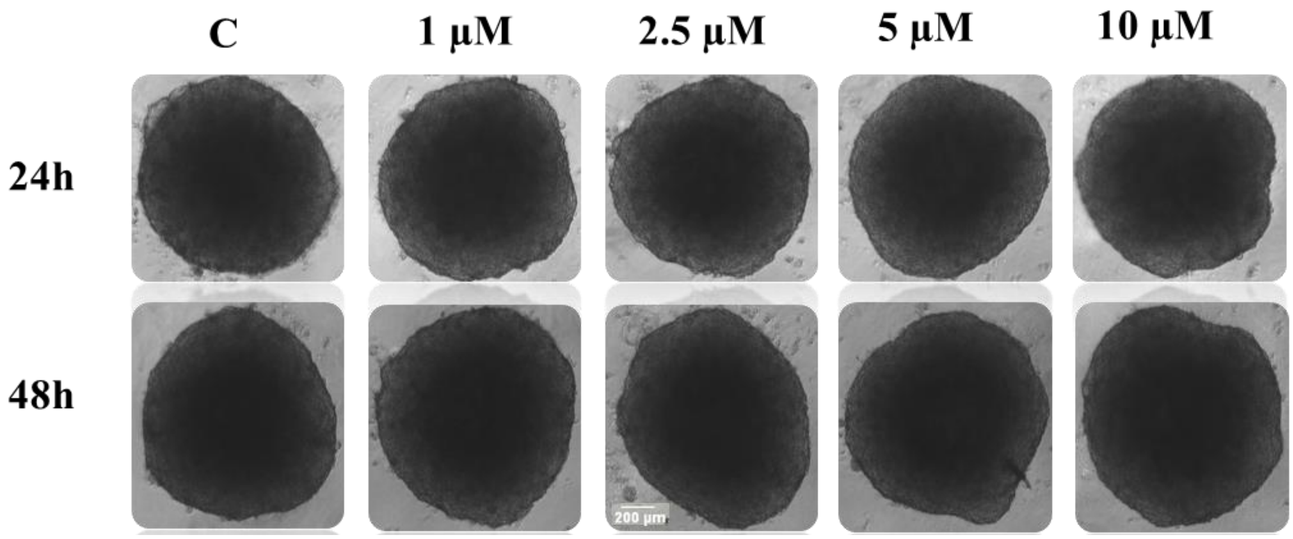

Figure. S2 The cytotoxic effects of CA-Mel on HT-29 spheroids. The spheroids were obtained after seeding 5000 cells/well and they were incubated for three days. On the third day of growth, the spheroids were treated with various concentrations of CA-Mel (1, 2.5, 5 and 10 µM). Images were taken with the help of light microscopy a 24 and 48 h, with the 4x objective. The scale bar is 200 µm and is identical for all the images.

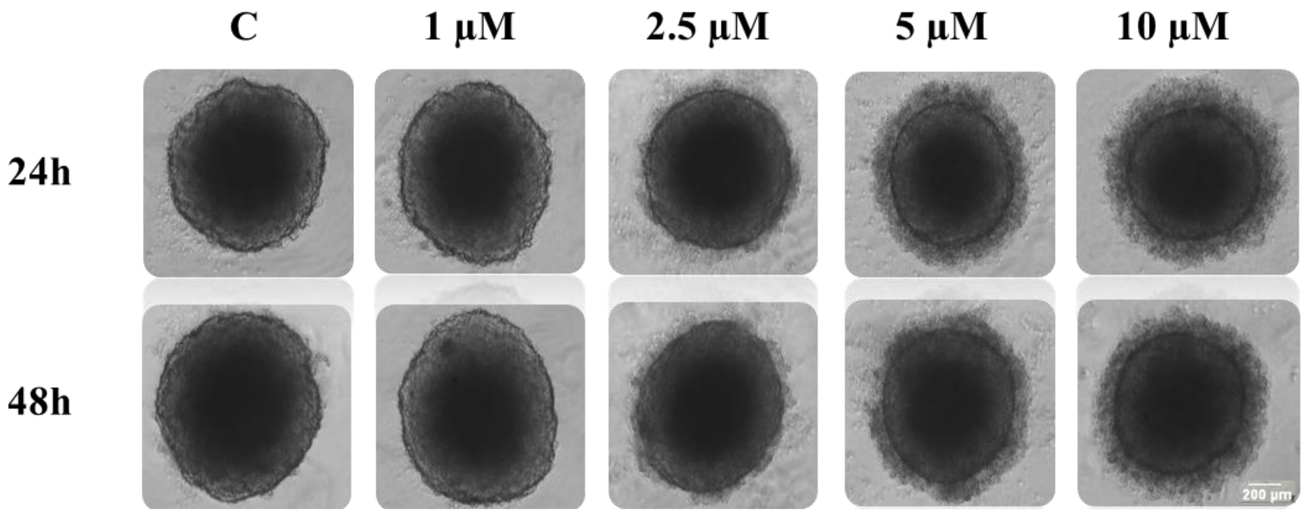

Figure. S3 The cytotoxic effects of Mel on HCT-116 spheroids. The spheroids were obtained after seeding 5000 cells/well and they were incubated for three days. On the third day of growth, the spheroids were treated with various concentrations of melittin (1, 2.5, 5 and 10 µM). Images were taken with the help of light microscopy a 24 and 48 h, with the 4x objective. The scale bar is 200 µm and is identical for all the images.

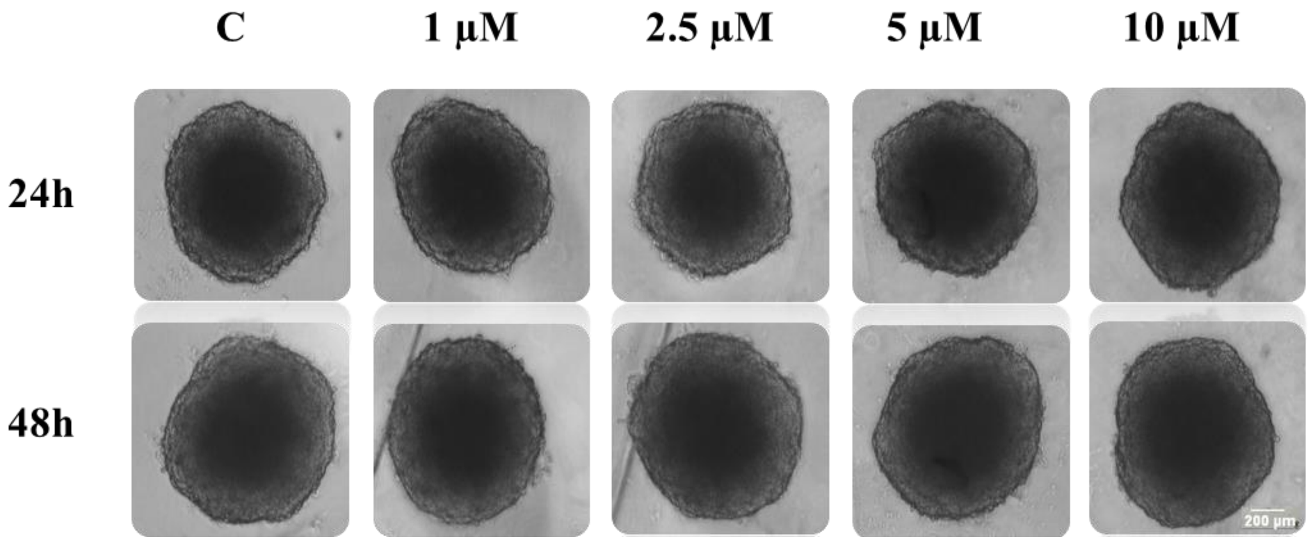

Figure. S4 The cytotoxic effects of CA on HCT-116 spheroids. The spheroids were obtained after seeding 5000 cells/well and they were incubated for three days. On the third day of growth, the spheroids were treated with various concentrations of CA (1, 2.5, 5 and 10 µM). Images were taken with the help of light microscopy a 24 and 48 h, with the 4x objective. The scale bar is 200 µm and is identical for all the images.

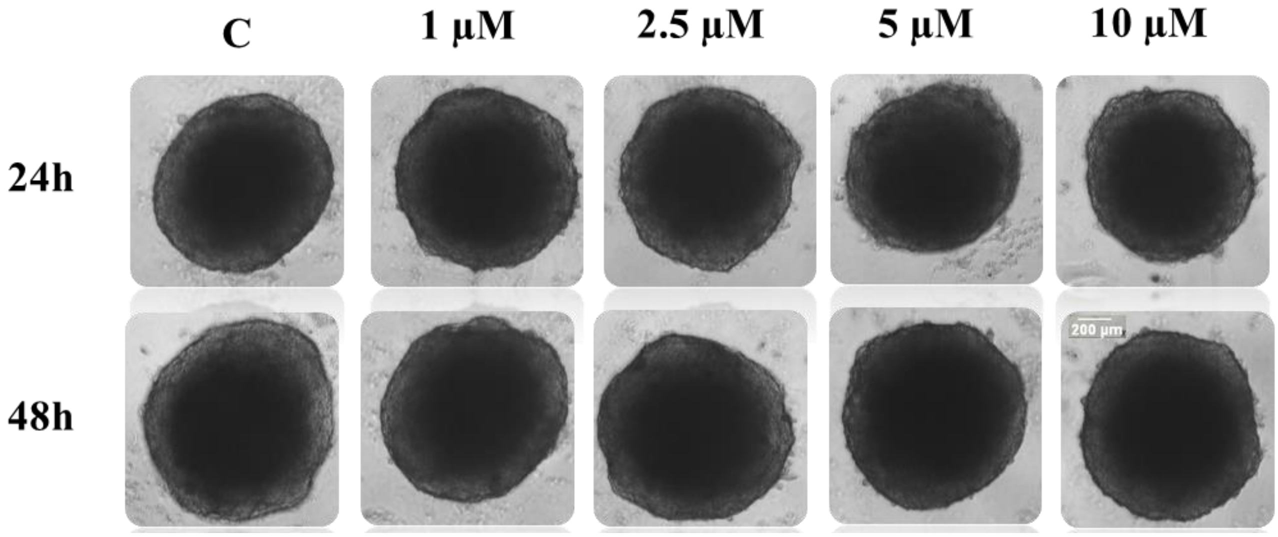

Figure. S5 The cytotoxic effects of CA-Mel on HCT-116 spheroids. The spheroids were obtained after seeding 5000 cells/well and they were incubated for three days. On the third day of growth, the spheroids were treated with various concentrations of CA-Mel (1, 2.5, 5 and 10 µM). Images were taken with the help of light microscopy a 24 and 48 h, with the 4x objective. The scale bar is 200 µm and is identical for all the images.
